# Exchange Bias in La_0.67_Sr_0.33_MnO_3_/YFeO_3_ Ferromagnet/Antiferromagnet Multilayer Heterostructures

**DOI:** 10.1002/smll.202501644

**Published:** 2025-04-13

**Authors:** Paul Fourmont, Eunsoo Cho, Sylvain G. Cloutier, Caroline A. Ross

**Affiliations:** ^1^ École de Technologie Supérieure Department of Electrical Engineering 1100 Notre Dame Street West Montreal Quebec H3C 1K3 Canada; ^2^ Massachusetts Institute of Technology Department of Materials Science and Engineering 77 Massachusetts Avenue Cambridge MA 02139 USA

**Keywords:** epitaxial thin films, exchange bias, multilayers, perovskite, spin‐valve

## Abstract

Exchange bias (EB), manifested as a hysteresis‐loop offset after field‐cooling, is demonstrated in perovskite‐structured ferromagnet/antiferromagnet (La_0.67_Sr_0.33_MnO_3_/YFeO_3_)_n_ heterostructures grown on (100) SrTiO_3_ substrates. Bilayer samples show an EB of 306 Oe at 50 K, whereas multilayers with five layers exhibit an exchange bias of up to 424 Oe at 50 K. A spin valve consisting of La_0.67_Sr_0.33_MnO_3_/SrTiO_3_/La_0.67_Sr_0.33_MnO_3_/YFeO_3_ shows stable remanent configurations resulting from pinning of the upper La_0.67_Sr_0.33_MnO_3_ layer by the YFeO_3_. In contrast, EB is not observed on (111)‐oriented SrTiO_3_ substrates due to interface roughening. These results demonstrate YFeO_3_ as an alternative orthoferrite antiferromagnet compared to BiFeO_3_ and LaFeO_3_ for incorporation into exchange‐biased heterostructures.

## Graphical abstract

Exchange bias is demonstrated in perovskite‐structured ferromagnet/antiferromagnet (La_0.67_Sr_0.33_MnO_3_/YFeO_3_)_n_ heterostructures grown on (100) SrTiO_3_ substrates, and a spin valve consisting of La_0.67_Sr_0.33_MnO_3_/SrTiO_3_/La_0.67_Sr_0.33_MnO_3_/YFeO_3_ shows stable remanent configurations resulting from pinning of the upper La_0.67_Sr_0.33_MnO_3_ layer by the YFeO_3_. These results demonstrate YFeO_3_ as an alternative orthoferrite antiferromagnet compared to BiFeO_3_ and LaFeO_3_ for incorporation into exchange‐biased heterostructures.

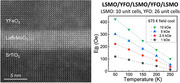

## 1 Introduction

Thin film heterostructures exhibit emergent interfacial phenomena that are absent in the individual constituents, making them interesting for both fundamental studies and device development.^[^ ^1^, ^2^, ^3^, ^4^, ^5^, ^6^, ^7^ ^]^ Perovskites, ABO_3_ where A and B represent cation sites, provide a particularly rich variety of bulk and interfacial behavior due to their wide range of possible cation compositions and structural distortions. They can exhibit a spectrum of magnetic properties including antiferromagnetism, half‐metallic ferromagnetism, colossal magnetoresistance, or spin‐glass behavior.^[^ ^8^, ^9^ ^]^

Exchange bias (EB) is an important interfacial effect that occurs between an antiferromagnet (AFM) and a ferro‐ or ferrimagnet (FM), leading to a shift in the hysteresis loop of the FM and stabilization of certain remanent configurations that are useful in sensors and other devices.^[^ ^10^, ^11^, ^12^, ^13^, ^14^ ^]^ Conventionally EB is established by field cooling when the Curie temperature (T_C_) of the FM exceeds the Néel temperature (T_N_) of the AFM.^[^ ^15^, ^16^ ^]^ Field cooling (FC) the system in a sufficiently high field from above T_N_ saturates the FM, which couples with and orients uncompensated spins at the AFM interface leading to EB. However, “unconventional” EB can also be found in AFM/FM systems with T_N_ > T_C_, including bilayer thin films of (Fe_0.1_Ni_0.9_)_80_B_20_/CoO, FeMn/CuNi, NiO/Ni_0.75_Cu_0.25_, LaFeO_3_/La_0.7_Sr_0.3_MnO_3_, BiFeO_3_/La_0.7_Sr_0.3_MnO_3_ and CoCr_2_O_4_/Cr_2_O_3_ or in nanomaterials such as oxide‐coated Mn nanoparticles with the core being AFM and the shell FM.^[^ ^6^, ^15^, ^16^, ^17^, ^18^, ^19^ ^]^ Field cooling the AFM from above its T_N_ leads to a partial ordering of the AFM even though the FM is in a paramagnetic state.^[^ ^15^, ^16^ ^]^ Below the blocking temperature (T_B_), which is below the lower of T_N_ and T_C_, uncompensated spins from the AFM layer coupled with the FM layer lead to EB, manifested as a horizontal shift of the hysteresis loop and often an increase in coercivity.^[^ ^7^, ^9^, ^10^, ^11^ ^]^ The loop shift can be positive or negative depending on the FC process and the nature of the exchange interaction and vanishes above T_B_.^[^ ^7^, ^10^, ^13^ ^]^ EB is highly sensitive to the interface structural quality,^[^ ^7^, ^9^, ^10^, ^14^, ^20^, ^21^, ^22^ ^]^ and is inversely proportional to the FM layer thickness, with higher EB present in thin FM layers of a few unit cells (u.c.).^[^ ^11^, ^23^, ^24^ ^]^ In contrast, EB increases with AFM layer thickness until a saturation value is reached, often at AFM thicknesses of several tens of nanometers.^[^ ^5^, ^7^, ^10^ ^]^

Exchange coupling has been extensively studied in various combinations of perovskite bilayers consisting of FM La_0.67_Sr_0.33_MnO_3_ (LSMO) and an AFM layer such as BiFeO_3_, LaFeO_3_, SrMnO_3_, LaMnO_3_, SrRuO_3_, TbMnO_3_, YMnO_3_ or Eu_0.45_Sr_0.55_MnO_3_.^[^ ^5^, ^6^, ^8^, ^9^, ^11^, ^12^, ^13^, ^19^, ^20^, ^22^, ^24^, ^25^, ^26^, ^27^, ^28^ ^]^ Bilayers based on LSMO coupled with paramagnetic or FM perovskite layers such as LaNiO_3_ or La_0.67_Ca_0.33_MnO_3_ also demonstrated weak EB at low temperatures.^[^ ^29^, ^30^, ^31^ ^]^ In LSMO, electron hopping between adjacent Mn^3+^ (d^4^) and Mn^4+^ (d^3^) cations through O^2−^ anions is responsible for metallic transport and FM via double‐exchange.^[^ ^32^, ^33^, ^34^, ^35^ ^]^ Depending on the deposition parameters and film thickness, T_C_ of LSMO films can reach 370 K, and LSMO possesses useful properties including half‐metallicity and colossal magnetoresistance which have been used in spin valves.^[^ ^36^, ^37^ ^]^ In contrast, orthoferrites such as LaFeO_3_ (LFO), YFeO_3_ (YFO) or LuFeO_3_ and rhombohedral BiFeO_3_ (BFO) are canted G‐type AFMs as a result of superexchange coupling between the Fe^3+^ (d^5^) cations, leading to a high T_N_ of ≈640 K for YFO and BFO.^[^ ^6^, ^38^, ^39^, ^40^, ^41^, ^42^ ^]^ BFO is a well‐known multiferroic with a ferroelectric polarization arising mainly from the Bi lone pairs.^[^ ^43^ ^]^ Bulk orthoferrites lack ferroelectricity, but ferroelectric behavior has been demonstrated in Y‐rich YFO (also in Lu‐rich LuFeO_3_) films due to structural distortions around Y_Fe_ antisite defects, making the material multiferroic.^[^ ^44^, ^45^ ^]^

Among the FM manganate perovskites, LSMO has one of the highest T_C_ making it a candidate for room‐temperature spintronics applications.^[^ ^36^, ^46^, ^47^ ^]^ On the contrary, AFM manganate perovskites such as LaMnO_3_, CaMnO_3_, PrMnO_3_, NdMnO_3_, SmMnO_3_, BaMnO_3_, and SrMnO_3_ possess a T_N_ between 100 and 150 K,^[^ ^5^, ^48^ ^]^ therefore manganate perovskite heterostructures only exhibit EB at low temperatures. The majority of work on EB in LSMO has been carried out on LSMO/BFO heterostructures which exhibit unconventional EB.^[^ ^5^, ^6^, ^20^, ^21^, ^22^, ^27^, ^28^, ^39^, ^49^, ^50^ ^]^ A substantial magnetic moment has been reported in the BFO layer, larger than the small bulk canted moment of BFO, and the LSMO and BFO moments are exchange‐coupled parallel or antiparallel depending on the sequence of ionic layers at the interface and the amount of intermixing.^[^ ^20^, ^21^, ^22^, ^28^, ^39^, ^49^, ^50^ ^]^ In contrast, LSMO/LFO shows spin flop coupling in which the spins in the two layers are orthogonal.^[^ ^11^, ^19^ ^]^ EB in LSMO/LFO of 260 and 120 Oe was found for 8 u.c. and 10 u.c. thick LSMO layers, respectively but the 10 u.c. thick LSMO sample showed a 50 K higher blocking temperature.^[^ ^11^ ^]^

In this article we report EB in La_0.67_Sr_0.33_MnO_3_/YFeO_3_ (LSMO/YFO) bilayers and multilayers grown on SrTiO_3_ (STO) substrates, showing that YFO is capable of exchange‐biasing LSMO. Most work on EB in perovskite heterostructures has focused on bilayers, yet EB can be enhanced in metal or metal‐ceramic multilayers compared to bilayers,^[^ ^51^, ^52^, ^53^, ^54^ ^]^ motivating our study of EB in perovskite multilayers. For LSMO/YFO heterostructures we describe the effect of thickness of both the FM and AFM layers on EB and T_B_, and obtain an EB of 306 Oe at 50 K for a bilayer of LSMO/YFO with thicknesses of respectively 10 and 49 u.c., and a T_B_ just below the T_C_ of the LSMO film. We show that multilayer structures have higher EB than bilayers, reaching 424 Oe at 50 K for a five‐layer LSMO/YFO/LSMO/YFO/LSMO stack. EB is proportional to the applied field during cooling, and its sign matches the direction of the applied field while cooling the system from above T_N_, i.e., a positive EB^[^ ^55^ ^]^ Finally, we design a spin‐valve LSMO/STO/LSMO/YFO to demonstrate independent switching of the free and pinned magnetic layers.

## 2 Results and Discussion

### 2.1 Structural Characterizations of LSMO/YFO Heterostructures

**Figure 1:**
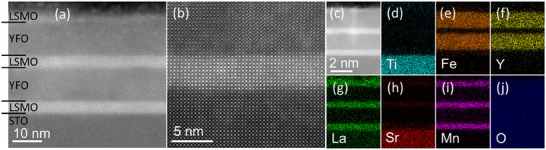
a) Cross‐section TEM image of a 32 nm thick sample made of successive LSMO and YFO layers on an STO substrate from CrysTec. b) High‐resolution TEM image of STO/LSMO/YFO layers. c) Cross‐section TEM image. (d‐j) EDS elemental maps of the image are shown in (c).

The bilayers and multilayers were deposited using pulsed laser deposition (PLD) onto (100)‐oriented STO substrates (Methods). Bilayers consisted of LSMO as the first layer with a thickness 3–5.5 nm (8–14 u.c.) and YFO as the upper layer with a thickness 3–19 nm (8–49 u.c.). **Figure** **1** illustrates the structural quality and composition analyzed using transmission electron microscopy (TEM) with energy‐dispersive X‐ray spectroscopy (EDS) elemental mapping for a multilayer sample with a total of five LSMO (10 u.c.) and YFO (26 u.c.) layers with a combined thickness of 32 nm. In Figure 1a,b, the atomic number (Z)‐contrast leads to layers with lighter elements (STO) appearing darker than those with heavier elements (LSMO).^[^ ^9^ ^]^ EDS elemental maps, Figure 1d–j qualitatively show the expected distribution of elements, with a composition profile given in Figure S1a (Supporting Information). High‐resolution TEM and EDS elemental maps of the STO/LSMO/YFO layers, Figure S1c–e (Supporting Information), indicate an interfacial roughness less than 1 u.c. To assess the effect of FC on the multilayer structure, we compared TEM and EDS mapping for two samples that were FC from 673 K one time (Figure S2, Supporting Information) and ten times (Figure 1). The sample with additional FC cycles did not show any greater interface widths, so we conclude that annealing up to 673 K does not measurably influence the structure and composition of the multilayer. On the contrary, temperatures ≈373 K can be detrimental to exchange bias in bilayers consisting of AFM perovskites coupled with metallic FM layers such as LaFeO_3_/Fe or BFO/CoFe systems due to chemical interdiffusion.^[^ ^42^, ^56^ ^]^

Figure S3 (Supporting Information) confirms the epitaxial growth by X‐ray diffraction. The peaks from the LSMO and YFO overlap, and no other phases are detectable. The peak position, with 2θ value ≈1.4° higher than that of the substrate (002) peak, is similar to that of our prior work on YFO.^[^ ^45^ ^]^ Considering the orthorhombic bulk YFO unit cell (a = 5.587 Å, b = 5.274 Å, c = 7.595 Å) the pseudocubic unit cell volume of (3.824 Å)^3^ of YFO is smaller than that of STO (lattice parameter of 3.905 Å) and the YFO film is under tensile in‐plane strain on an STO substrate. LSMO with a bulk lattice parameter of 3.876 Å is also under tensile in‐plane strain (lattice mismatch of 0.75%).^[^ ^57^, ^58^ ^]^

Previous studies on LSMO reported that a critical film thickness is necessary to obtain magnetic ordering and suppress magnetic dead layers,^[^ ^36^ ^]^ typically 1.2–5 nm for LSMO/STO.^[^ ^6^, ^11^, ^32^, ^36^, ^47^ ^]^ Magnetization depth profiles calculated from nuclear scattering length density measurements on LSMO/STO samples showed that magnetization is maximum at the substrate interface and decreases toward the surface of the film when its thickness exceeds 5 nm.^[^ ^47^ ^]^ Hence, the thickness of the LSMO layer should be optimized to suppress magnetic dead layers while keeping it thin enough to maximize its magnetic properties and its coupling with the AFM layers.^[^ ^47^ ^]^ In addition, T_C_ is reduced if the LSMO film is fully strained and shows a strong dependence on LSMO thickness, increasing from 100 to 350 K for 5–26 u.c. respectively.^[^ ^36^, ^47^ ^]^ We measured T_C_ = 280 ±  2 K for a 10 u.c. single layer of LSMO (Figure S4a, Supporting Information) which is similar to previous work.^[^ ^2^, ^5^, ^36^ ^]^

Figure S4c (Supporting Information) shows that the saturation magnetization M_S_ of the 10 u.c. LSMO layer varies between 126 ±  6 and 354 ±  18 emu.cm^−3^ (based on total film thickness) and its coercivity H_C_ from 38 to 43 ±  1 Oe at 250 and 50 K respectively. Both M_S_ and H_C_ are consistent with the ranges of values shown in previous works, which report a dependence of H_C_ on film thickness and a dependence of M_S_ on film thickness and on deposition parameters such as the oxygen pressure.^[^ ^36^, ^59^ ^]^ In Figure S4c (Supporting Information), H_C_ remains almost constant with temperature and M_S_ increases with decreasing temperature. Figure S4a (Supporting Information) shows a slope change at 105 K in the magnetization (measured at 100 Oe) versus temperature. This temperature corresponds to a structural change in the STO substrate from cubic to tetragonal,^[^ ^60^ ^]^ which changes the strain state and anisotropy of the LSMO and hence its low field magnetization. LSMO/YFO heterostructures did not show an effect of the structural change in their M(T) measured at 100 Oe.

### 2.2 Exchange Bias in Bilayers and Multilayers of LSMO/YFO

**Figure 2:**
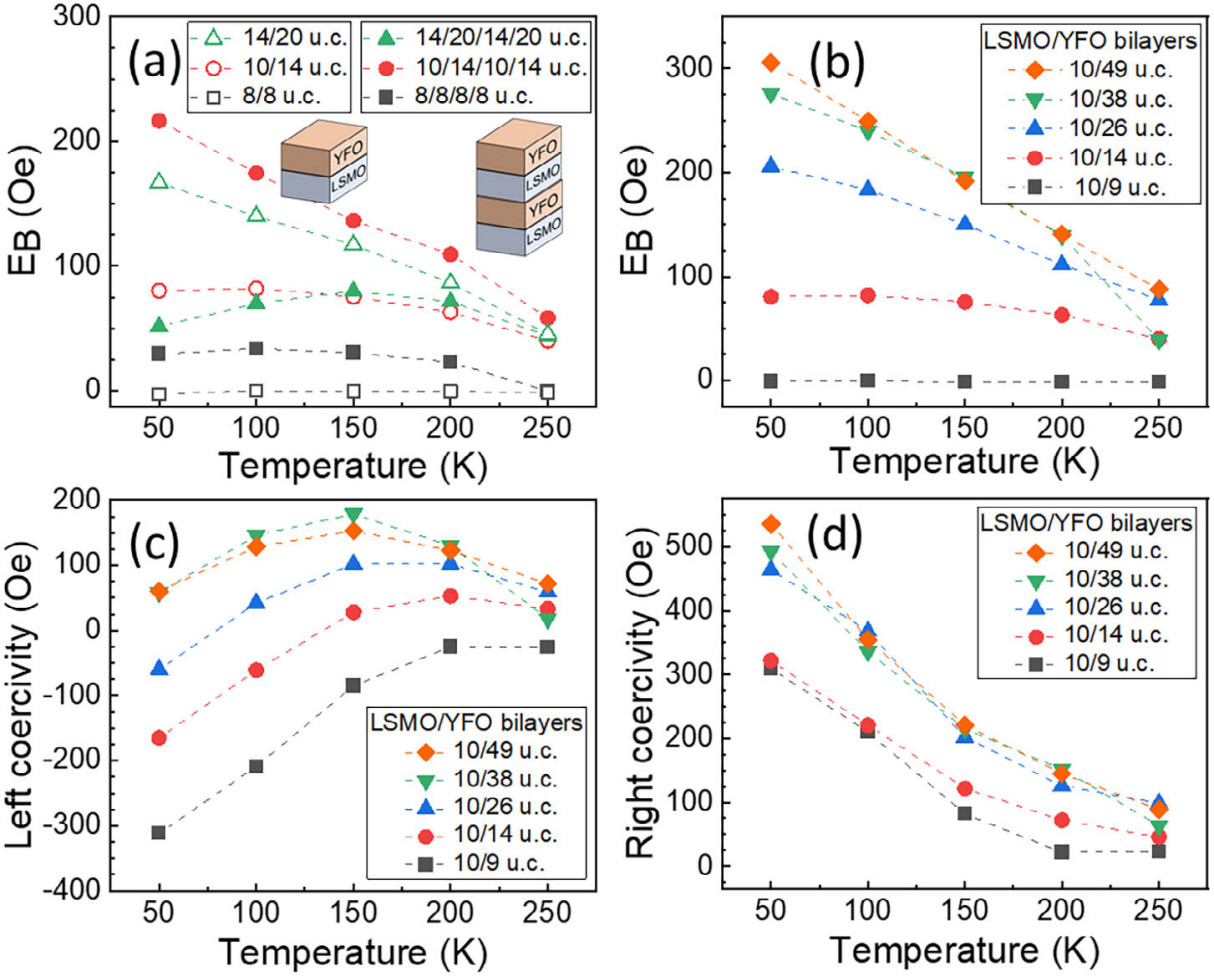
a) Exchange bias versus temperature of bilayers and four‐layer samples for different LSMO/YFO layer thicknesses deposited on CrysTec STO substrates. b) Effect of different YFO thickness using a fixed LSMO thickness of 10 u.c. c,d) Left and right coercivity of the bilayers shown in (b). The dashed lines serve as a guide to the eye.

**Figure** **2** shows the EB at different temperatures for LSMO/YFO heterostructures with different layer sequences and layer thicknesses, after FC at 10 kOe from 673 K (i.e., above T_N_). EB is calculated using the formula H_EB_ = (H^+^+H^−^)/2 where H^+^ and H^−^ are the coercivities, i.e., the fields where the hysteresis loop crosses the field axis in the ascending (right coercivity) and descending (left coercivity) branches of the FC hysteresis loop, respectively. When EB is present it is positive, i.e., |H^+^| > |H^−^|. As a control, FC of a 10 u.c. LSMO layer without YFO yielded the expected zero EB. The canted magnetization of the YFO is 0.05 emu.g^−1^,^[^ ^61^ ^]^ and it cannot be distinguished from the LSMO signal in the hysteresis loops.

Figure 2a compares LSMO bilayers and 4‐layer films. Increasing the layer thicknesses from the 8/8 u.c. LSMO/YFO bilayer (8 u.c. = 3.1 nm) to the 10/14 and 14/20 u.c. bilayers raised the EB, which also increased at lower temperatures. Increasing from two to four layers raised the EB in some cases: the 8/8 u.c. sample had no EB at 200 K whereas the 8/8/8/8 u.c. sample shows a small EB of 23 ± 1 Oe at 200 K. The 10/14/10/14 u.c. sample has an EB = 110 ±  1 Oe, about twice that of the 10/14 u.c. sample. However, the thickest film did not follow this trend: the 14/20/14/20 u.c. structure had a lower EB than its bilayer counterpart, possibly indicating degradation of the layer structure in the thicker film due to roughening. The 10/14 and 14/20 u.c. bilayers and the 10/14/10/14 u.c. and 14/20/14/20 u.c. four‐layer films all show T_B_ > 250 K.

From Figure 2b, EB increases with YFO film thickness for a fixed LSMO film thickness of 10 u.c., saturating above 38 u.c. (14.8 nm). This saturation behavior resembles that of LSMO/BFO bilayers where the EB saturates for BFO thickness above 25 nm.^[^ ^5^ ^]^ In contrast, EB decreases with increasing LSMO thickness (not shown). A 25/29 u.c. LSMO/YFO bilayer showed no EB even at 50 K, compared to the 10/26 u.c. sample from Figure 2b which shows EB at 50–250 K. This indicates that thick LSMO is not effectively pinned by YFO, and is consistent with previous work reporting EB in LSMO/LFO bilayers only for LSMO thicknesses below 20 u.c.^[^ ^11^ ^]^ Compared to bilayers of LSMO/BFO which possess a T_B_ ≈ 110 K and an EB ≈ 25 Oe at 50 K, LSMO/YFO bilayers possess superior properties with a T_B_ ≈ 280 K and an EB ≈ 306 Oe at 50 K.^[^ ^5^, ^27^, ^28^ ^]^ Layer sequence can affect the composition profile and structure of the interface, as well as the strain state which influences T_C_ and M_s_ as described for SrRuO_3_/SrMnO_3._^[^ ^62^ ^]^ Here we found that inverting the order of the layers, i.e., growing YFO under the LMSO, yielded a lower EB as shown in Figure S5 (Supporting Information).

Figure 2c,d shows the evolution of the left (descending branch, H^−^) and right (ascending branch, H^+^) coercivities of bilayers made with LSMO layers of 10 u.c. and with increasing YFO thickness from 9 to 49 u.c. The coercivities have different dependences on YFO thickness. The left coercivity becomes more positive with thicker YFO layers until reaching saturation at 38 u.c., similar to the EB in Figure 2b. In comparison, the right coercivity has little dependence on YFO thickness except for being lower at 9 u.c. YFO. Thus increased YFO thickness raises EB and reduces the loop width (H^+^‐H^−^)/2 as shown in Figure S3c (Supporting Information). This change is attributed to the left coercivity which moves to more positive values.

**Figure 3:**
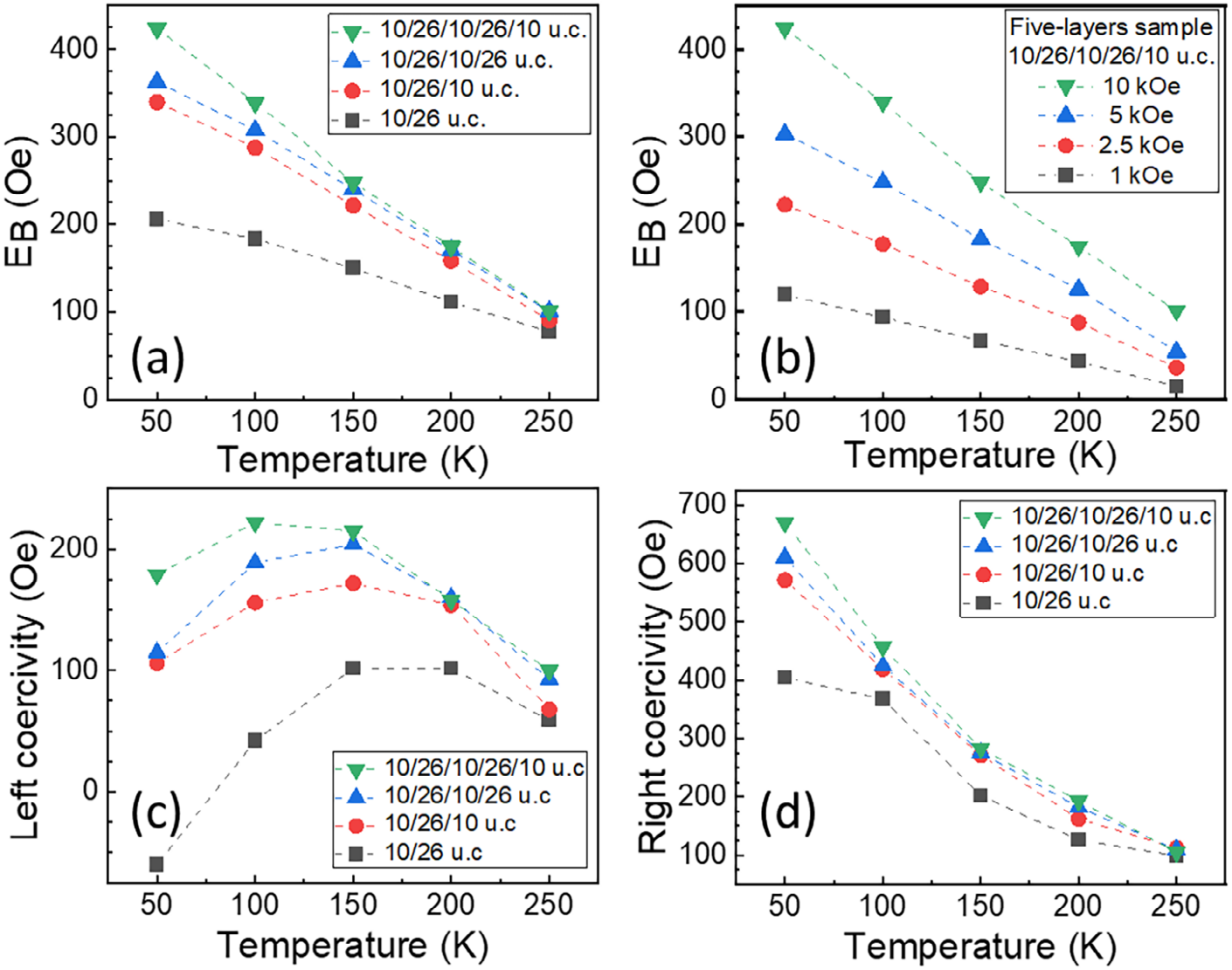
a) Exchange bias versus temperature for increasing number of LSMO and YFO layers deposited on CrysTec STO substrates. b) Effect of different fields on EB while cooling the sample from 673 K. c,d) Left and right coercivity of the multilayers shown in (a).

**Figure** **3**a reports the evolution of EB for stacks made with different numbers of layers of 10 u.c. LSMO and 26 u.c. YFO. EB increases with the number of layers with the largest differences at low temperatures. The five‐layer sample with a total thickness 32 nm exhibited the highest EB of 424 Oe at 50 K. Enhanced EB has been reported in metal and metal‐oxide multilayers,^[^ ^51^, ^52^, ^54^ ^]^ and this study shows that there is also an EB enhancement due to multilayering in all‐oxide heterostructures. Figure 3c,d presents the left and right coercivities measured from the descending and ascending branches respectively of the FC hysteresis loop, for the samples shown in Figure 3a. Both coercivities become more positive as the number of layers increases, exhibiting analogous trends to those seen in Figure 2c,d.

### 2.3 Training Effects From Field Cycling

**Figure 4:**
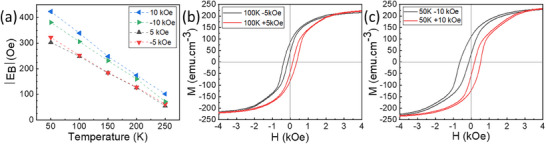
a) Influence of different fields on the magnitude of EB on the 32 nm thick five‐layer sample shown in Figure 1 cooled from 673 K. Negative fields result in negative EB values. b,c) hysteresis loops obtained at various temperatures and fields.

**Figures** **4** and S6 (Supporting Information) illustrate training effects on EB for structures with 10 u.c. LSMO. The effect of multiple positive FC cycles is shown by comparing Figure S6a (Supporting Information) with Figure 2b. A second cycle led to an increase in EB from 206 to 356 Oe at 50 K for the 10/26 u.c. bilayer (and a smaller increase for the 10/38 u.c. bilayer), while EB remained constant for the 10/49 u.c. bilayer. Such behavior has been explained as a result of the lower anisotropy energy in the thinner (26 or 38 u.c.) AFM layer.^[^ ^15^ ^]^ EB also increased with the field applied during the FC process, as shown in Figure S6b (Supporting Information) for the 10/26 u.c. bilayer FC from 370 K (i.e., above T_C_ and below T_N_). Similar measurements are presented for a five‐layer sample (10/26/10/26/10 u.c.) while cooling from 673 K in Figure 3b and from 370 K in Figure S6c (Supporting Information). Due to equipment constraints, a maximum field of 10 kOe was possible in a FC from 673 K whereas 70 kOe could be applied while cooling the sample from 370 K. Both FC processes yielded EB of up to 425–450 Oe measured at 50 K. This shows that EB increases with both field and temperature applied during FC. Zn_0.7_Ni_0.3_Fe_2_O_4_/BFO bilayers also showed lower EB when the FC temperature was reduced.^[^ ^63^ ^]^ While field cooling from above T_N_, the AFM layer will undergo ordering while the FM layer is still above its T_C_ and is in a paramagnetic state. Field cooling the sample from below T_N_ but above T_C_ induces a small magnetization in the LSMO layer and a small canted magnetization in the AFM layer which interacts to give EB. We expect that a higher field would be needed to obtain the same EB if we start the field cooling from below T_N_, because the susceptibility of the AFM is higher at T_N_.

Figure S6c,d (Supporting Information) demonstrates that the sequence of the measurements while cooling the sample from 370 K impacts EB. (The samples used in Figure S6c,d (Supporting Information) were made with the same layers but on substrates from different vendors.) In Figure S6c,d (Supporting Information) the FC used field sequences of 0/70/10/30/20 and 0/10/20/30/70 kOe respectively. The former sequence shows that applying the 70 kOe field first enables retention of EB for successively lower fields, with a variation of EB of ≈50 Oe. On the contrary, a progressive increase of the field during FC from 370 K results in a monotonic increase of EB, Figure S6d (Supporting Information). In both cases, the largest field of 70 kOe yields the highest EB. Therefore EB can be achieved without a high‐temperature process, making the LSMO/YFO potentially integrable onto heat‐sensitive or flexible substrates.

For the 10/26/10/26/10 u.c. five‐layer sample FC from 673 K, Figure 4a,b shows that the magnitude of the EB is similar for ‐5 and 5 kOe fields, with negative fields resulting in reversal of the EB values. However, the magnitude of the EB for the −10 kOe field is lower than that obtained from the +10 kOe field, respectively: 383 and 424 Oe at 50 K as shown in Figure 4c. Since measurements were made sequentially on the same sample, this likely results from a training effect.

### 2.4 All‐Perovskite Spin‐Valves

The EB that we have demonstrated motivates the development of an all‐perovskite spin valve structure. The tunneling magnetoresistance of LSMO/STO/LSMO trilayers has been reported for LSMO thicknesses varying from 0.28 to 3.9 nm,^[^ ^37^, ^64^, ^65^, ^66^, ^67^ ^]^ for example LSMO/STO (2.8 nm)/LSMO yielded magnetoresistance ratios of 1850% at 4.2 K and 12% at 270 K.^[^ ^64^ ^]^ Other oxide spin valve examples are based on spinel ferrites.^[^ ^63^, ^64^, ^65^ ^]^ Zn_0.7_Ni_0.3_Fe_2_O_4_/LaNiO_3_/Zn_0.7_Ni_0.3_Fe_2_O_4_/BFO on (100) STO showed a RT magnetoresistance ratio of 0.8% and bilayers of BFO/Zn_0.7_Ni_0.3_Fe_2_O_4_ showed an EB of 20 Oe at RT,^[^ ^63^ ^]^ while a Fe_3_O_4_/MgO/Fe_3_O_4_/NiO stack on MgO demonstrated exchange coupling between the Fe_3_O_4_ layers.^[^ ^68^ ^]^

To demonstrate a heterostructure with both pinned and unpinned layers, we synthesized an all‐perovskite spin‐valve stack consisting of STO/LSMO(10 u.c.)/STO(8 u.c.)/LSMO(10 u.c.)/YFO(26 u.c.), and FC the sample at 10 kOe and 673 K. The hysteresis loops measured at 150 and 200 K (Figure S7, Supporting Information) exhibit a characteristic two‐step switching attributed to the magnetic reversal of the free (lower) and pinned (upper) LSMO layers,^[^ ^14^, ^69^ ^]^ which are decoupled by the STO interlayer. At 200 K, parallel and antiparallel remanent states can be achieved, with each layer having a coercivity of 30–40 Oe. The EB of the pinned layer is 40 Oe, hence the remanent state after positive saturation consists of antiparallel pinned and free layers. The EB is less than that measured for a 10/26 u.c. bilayer after the same FC process (Figure 2b) which may originate from greater roughness for the pinned layer grown onto the free layer/STO spacer.

For our LSMO/YFO bilayers, multilayers, and spin valves the EB is always in the same direction as the field applied during FC (Figure 4), i.e., a positive EB. Positive EB is rare, with most FM/AFM interfaces exhibiting negative EB, but it has been reported in heterostructures such as LaSrFeO_3_/SrRuO_3_ with a mosaic texture.^[^ ^55^ ^]^ Positive EB has been attributed to a change in the interfacial charge distribution which influences interfacial charge transfer,^[^ ^70^ ^]^ and hence magnetic properties of LSMO.^[^ ^71^ ^]^ The surface termination at the interfaces also affects polar discontinuities and charge reconstructions which influence the interface coupling.^[^ ^20^ ^]^ The presence of EB suggests that FC from above T_N_ creates a net uncompensated moment in the AFM YFO layer that pins the LSMO layer. Considering the energetic deposition process, and the evidence of Figure S1 (Supporting Information) that the interfaces are not atomically abrupt, this result may be analogous to the AFM coupling reported between LSMO and the uncompensated interfacial region of BFO in LSMO/BFO with intermixed interfaces.^[^ ^21^, ^22^, ^50^ ^]^ It contrasts with LSMO/YMnO_3_ bilayers with negative EB, attributed to an FM coupling due to Mn double‐exchange coupling.^[^ ^13^ ^]^ Positive and negative EB are often attributed to FM and AFM coupling respectively,^[^ ^11^, ^70^ ^]^ but in BFO/LSMO heterostructures with negative EB, both FM and AFM couplings have been found depending on the surface termination.^[^ ^20^ ^]^

### 2.5 Effect of the Substrate Orientation on EB

**Figure 5:**
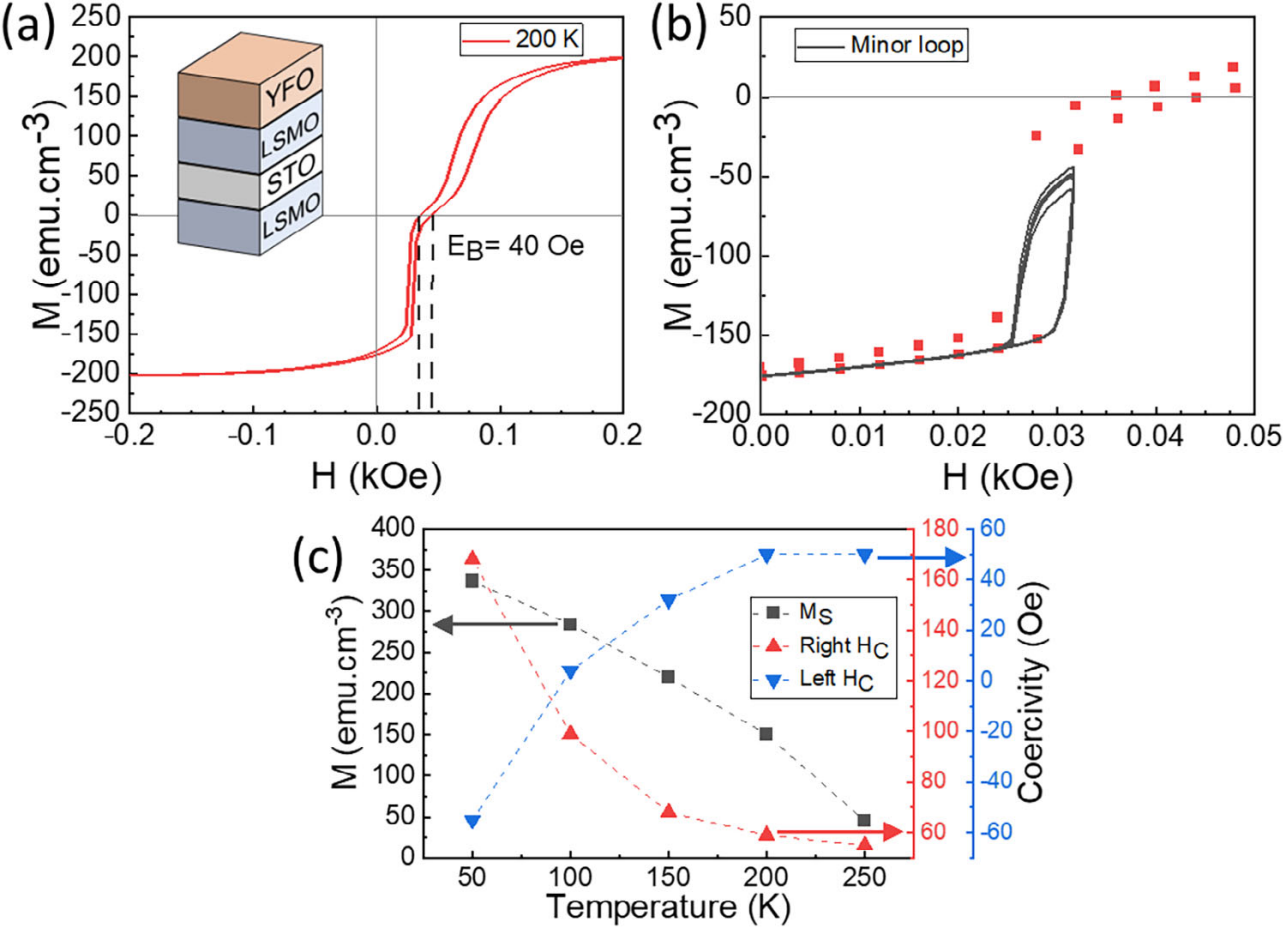
a) Minor hysteresis loops of the LSMO(10 u.c.)/STO(8 u.c.)/LSMO(10 u.c.)/YFO(26 u.c.) multilayer spin‐valve sample grown on CrysTec (100) STO substrate at 200 K after FC at 10 kOe and 673 K. b) inset of Figure (a) with 5 minor loops, c) Evolution of the magnetization and the coercivity of the minor loops with temperature.

**Figure 6:**
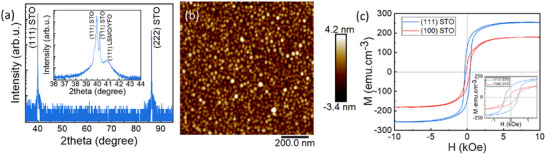
a) X‐ray diffraction pattern of the five‐layer sample deposited on (111) STO supplied by MTI, with inset showing the (111) peaks b) AFM image of the (111) sample. c) magnetic hysteresis loops of samples on both (111) and (100) STO from MTI at 50 K after FC. The inset shows the shift of the hysteresis curve for the (100) stack compared to the (111).

The (111) (pseudocubic) planes of the G‐type AFM are uncompensated whereas (100) are compensated, suggesting that YFO/LSMO grown on (111)‐oriented STO may be expected to have an enhanced EB compared to the (100) orientation. To examine orientation effects, the optimized five‐layer heterostructure of the sample in Figure 1 and the spin‐valve stack of the sample in **Figure** **5** were re‐grown on both (100) and (111) STO substrates. All the samples were then FC at 10 kOe from 673 K. The growth rate was lower for the re‐deposited samples (five‐layer stack in **Figure** **6** and spin‐valve in Figure S8, Supporting Information) compared to the initial samples (five‐layer stack in Figure 1 and spin‐valve in Figure 5) leading to thinner LSMO layers with 10% lower M_s_. Figure 6a confirms epitaxial growth of the five‐layer sample on the (111) STO substrate.

Figure 6c shows that the five‐layer film on (111) STO had a higher M_S_ than the one on (100) STO, 255 versus 176 emu.cm^−3^ at 50 K, consistent with prior work of LMO/STO.^[^ ^47^ ^]^ The five‐layer sample on (100) STO did exhibit EB of 34–98 Oe between 250 and 50 K, Figure 6c. These values are smaller than those shown in Figure 3, due to the lower LSMO thickness. In contrast, the five‐layer sample on (111) STO did not show any EB from 250 to 50 K. AFM measurements yielded a r.m.s. roughness (R_q_ = 1.10 nm) that was four times higher compared to the multilayer grown on (100) STO. It is possible that the rougher interfaces are responsible for the lack of EB, but the relationship between interface roughness and EB remains under debate.^[^ ^7^, ^10^ ^]^ For example, in LSMO/BFO bilayers, higher roughness was associated with greater EB^[^ ^20^, ^22^ ^]^ whereas in FeF_2_/Fe bilayers higher roughness reduced EB^[^ ^72^ ^]^ and in MnF_2_/Fe bilayers roughness led to a change from negative to positive EB.^[^ ^73^ ^]^ The lack of EB in our (111)‐oriented films could also be a result of different surface terminations resulting in different net interfacial charges.^[^ ^47^ ^]^

Figure S8a,b,d,e (Supporting Information) compares the magnetic hysteresis loops of the FC spin‐valve stacks. The (100)‐oriented spin valve clearly showed different coercivities of the LSMO layers indicating exchange‐decoupling by the STO interlayer, but the sample did not show EB. This is attributed to the lower deposition rate which yielded thinner LSMO layers and hence reduced EB (compare Figure 2a). The (111)‐oriented spin valve showed a slower approach to saturation and switching of the two layers could not be distinguished. This behavior, and the lack of EB, is attributed to the increased roughness, with R_q_ = 1.85 nm for the spin‐valve stack deposited on (111) STO versus R_q_ = 0.34 nm for the spin‐valve on (100) STO, Figure S8c,d (Supporting Information). The lower roughness of (100)‐oriented perovskite films is due to their anisotropic surface energy, which favors (100)‐terminated surfaces.^[^ ^74^ ^]^ The roughening counteracts any benefit to the EB that may arise from the uncompensated pseudocubic (111) interface of the G‐type AFM.

## 3 Conclusion

Unconventional exchange bias is reported in all‐perovskite LSMO/YFO ferromagnet/antiferromagnet heterostructures deposited on (100)‐oriented STO substrates where the Curie temperature of the LSMO is lower than the Néel temperature of the YFO. The pulsed laser‐deposited heterostructures grow epitaxially with in‐plane tensile strain and interfacial roughness below 1 unit cell. FC performed from above or from below the YFO Néel temperature leads to a shift of the magnetic hysteresis loop in the same direction as the applied field resulting in a positive EB, unlike the negative EB found at most FM/AFM interfaces. FC from below the Néel temperature requires higher fields to yield the same EB compared to FC from above the Néel temperature.

The EB increases with decreasing temperature and with increasing YFO thickness. A LSMO 10 u.c./YFO 49 u.c. bilayer exhibits an EB of 306 Oe at 50 K with a Curie temperature ≈280 K. Increasing the number of layers in the stack enhances the EB, with a five‐layer stack showing an EB of 424 Oe at 50 K which is among the highest values reported so far for an all‐oxide FM/AFM. A spin‐valve multilayer was fabricated using STO as the spacer layer, which showed separate switching of the pinned and unpinned layers, and parallel and antiparallel remanent states. EB was not found in FC stacks deposited on (111)‐oriented STO despite the uncompensated YFO surface, which is attributed to the roughening of the interfaces. Compared to other reports of perovskite bilayers, LSMO/YFO heterostructures possess a higher T_B_ ≈280 K and higher EB compared to LSMO/BFO or LSMO/LFO which possess a T_B_ ≈110 and 200 K, respectively. In addition, EB in LSMO/YFO bilayers at 50 K is one order of magnitude higher than LSMO/BFO bilayers reported in the literature. These results make YFO a useful option for introducing large positive exchange bias in perovskite heterostructures.

## 4 Experimental Section

The bilayer and multilayer samples were deposited by PLD using a KrF excimer laser (λ = 248 nm) with ≈1 J cm^−2^ fluence. All the films were deposited successively by ablating up to three stoichiometric LSMO, YFO, and STO targets with a 2.54 cm diameter and a thickness between 6 and 12 mm. The targets were obtained from Kurt J. Lesker and Plasmaterials and used as received. The repetition rate is 10 Hz and the O_2_ partial pressure is fixed to 75 mTorr during all the depositions. The setpoint temperature of the substrate holder was 900 °C and the substrate itself is ≈150 °C below this. All the films are deposited on 5 × 5 mm STO (100) substrates, with additional five‐layer and spin valve samples synthesized on STO substrates with a (111) orientation.

STO substrates from two providers, MTI Corp. (USA) and CrysTec Kristalltechnologie (Germany) were used to compare their effect on EB values. The highest EB values are systematically obtained while using the substrates from CrysTec Kristalltechnologie which possess the lowest surface roughness and no detectable residual contamination from the polishing steps. In Figure S9a (Supporting Information), x‐ray reflectivity measurements on the 32 nm five‐layer samples deposited on both substrates during the same PLD run validate that smoother interfaces are obtained from CrysTec substrates. The decrease of intensity of the reflected X‐rays is similar for both samples implying similar surface roughness of the films.^[^ ^75^ ^]^ The amplitude of the oscillations is higher from the sample deposited on the CrysTec substrate indicating lower interface roughness between both the substrate and the first LSMO layers and the other LSMO/YFO layers.^[^ ^75^ ^]^ AFM characterizations of bare STO substrates from both providers are shown in Figure S9b,c (Supporting Information). Some particles are visible in Figure S9c (Supporting Information) from the MTI substrates and were found in several batches of substrates. The CrysTec Kristalltechnologie did not exhibit particles. The r.m.s. roughness was 0.212 nm and 1.20 nm for (100) STO from CrysTec Kristalltechnologie and MTI respectively.

The TEM lamellae were prepared by a Velion FIB‐SEM focussed ion beam system. The STEM images were obtained on a Themis Z STEM working at 200 keV with a chromatic corrector. The EDS elemental mapping and quantification were collected with a Thermo Fisher Scientific Super‐X EDS detector. Magnetic hysteresis was measured using a Quantum Design MPMS3 SQUID magnetometer between 250 and 50 K. Field cooling at 673 K was done on a Digital Measurement System 7035B vibrating‐sample magnetometer under nitrogen atmosphere. All the measurements are performed with a field applied along the in‐plane crystallographic [110] axis and the diamagnetic moment was subtracted. Structural characterization by high‐resolution X‐ray diffraction was done using a Smartlab Rigaku high‐resolution diffractometer with Cu K_α1_ radiation (λ = 1.5406 Å) as an X‐ray source and an incident beam Ge‐(220) double‐bounce monochromator.

## Conflict of Interest

The authors declare no conflict of interest.

## Supporting information



Supporting Information

## Data Availability

The data that support the findings of this study are available from the corresponding author upon reasonable request.
