# Supplementary material for: Exchange Bias in La0.67Sr0.33MnO3/YFeO3 Ferromagnet/Antiferromagnet Multilayer Heterostructures
Source: Small. 2025 Apr 13;21(21):2501644. doi: 10.1002/smll.202501644 (PMC12105432; doi:10.1002/smll.202501644)
Supplement: Supplementary file 1 — Supporting Information [file SMLL-21-2501644-s001.pdf]

NANO · MICRO

small

Supporting Information

for *Small*, DOI 10.1002/smll.202501644

Exchange Bias in $La_{0.67}Sr_{0.33}MnO_3/YFeO_3$ Ferromagnet/Antiferromagnet Multilayer Heterostructures

*Paul Fourmont, Eunsoo Cho, Sylvain G. Cloutier and Caroline A. Ross**

Exchange bias in $La_{0.67}Sr_{0.33}MnO_3/YFeO_3$ ferromagnet/antiferromagnet multilayer heterostructures

Paul Fourmont[a,b], Eunsoo Cho[b], Sylvain G. Cloutier[a] and Caroline A. Ross[b*]

[a] École de Technologie Supérieure, Department of Electrical Engineering, 1100 Notre Dame Street West, Montreal, Quebec H3C 1K3, Canada

[b] Massachusetts Institute of Technology, Department of Materials Science and Engineering, 77 Massachusetts Avenue, Cambridge, MA 02139, USA

* Corresponding author: caross@mit.edu

Supplementary Information

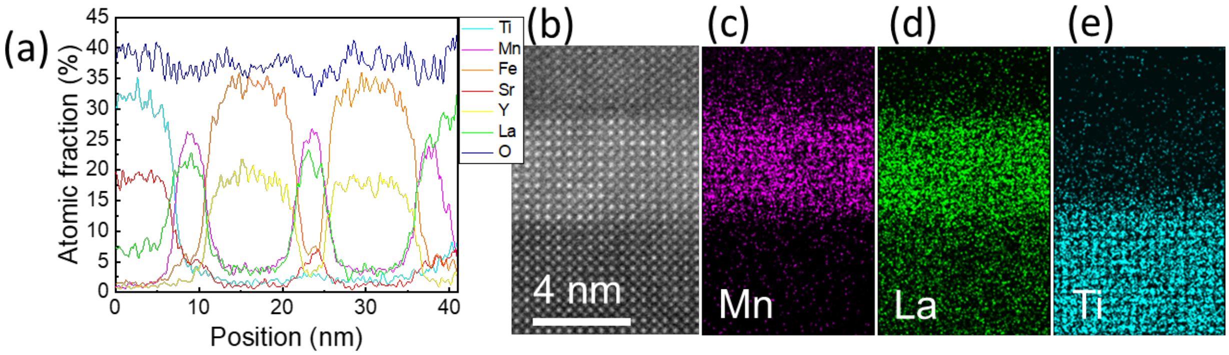

Figure S1 – (a) EDS line analysis through a cross-section image shown in Figure 1(a). (b) High-resolution cross-section TEM image of the STO/LSMO/YFO interfaces. (c-e) EDS elemental maps of the image shown in (b). This heterostructure is deposited on CrysTec STO.

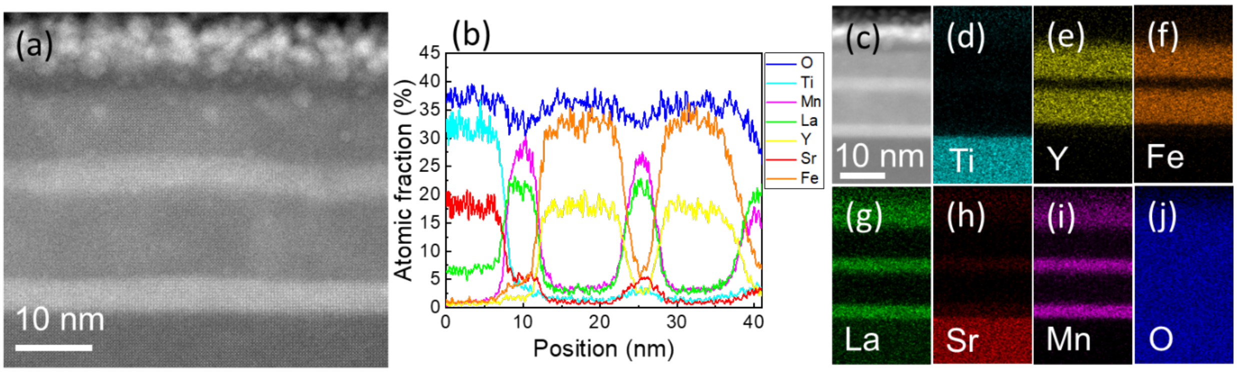

Figure S2 – (a) Cross section TEM image of a 32 nm thick sample made of successive LSMO and YFO layers on an MTI STO substrate. This sample has been field cooled only once. (b) EDS line analysis through a cross-section image shown in (a). (c) Cross-section TEM image. (d-j) EDS elemental maps of the image shown in (c).

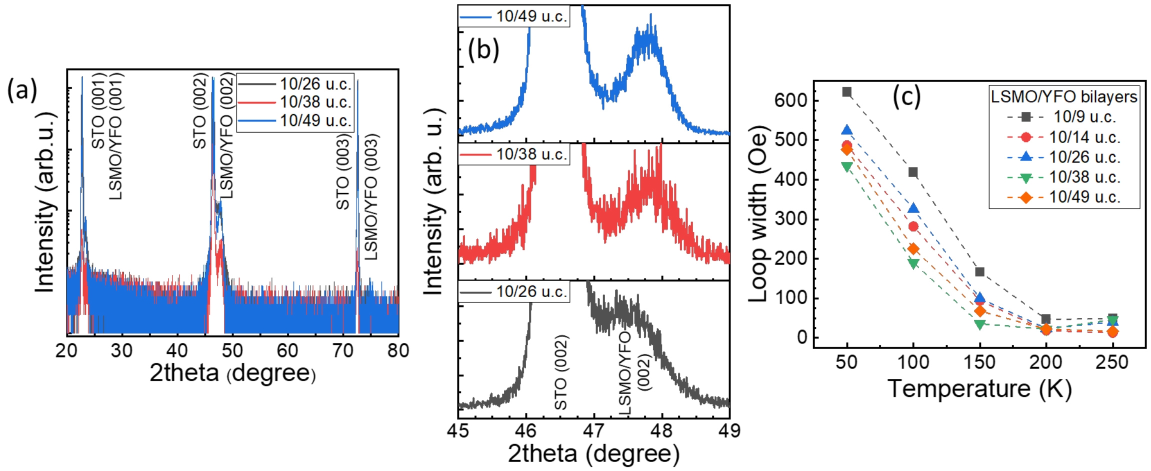

Figure S3 – (a-b) X-ray diffraction patterns of LSMO/YFO bilayers on CrysTec STO substrate. (b) Inset of the (002) peaks. (c) Evolution of the magnetic hysteresis loop width with the temperature for different thicknesses of YFO.

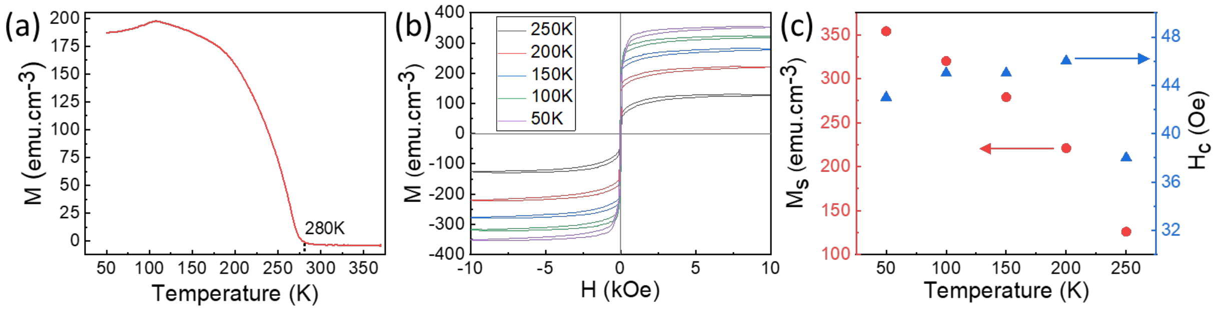

Figure S4 – Single layer of LSMO of 10 u.c. deposited on (100) CrysTec STO substrate (a) magnetization versus temperature curve measured at 100 Oe. The change in slope at 105 K is attributed to a structural transformation in the STO substrate which changes the strain state of the LSMO and its anisotropy, affecting the loop shape. (b) Magnetic hysteresis loops and (c) evolution of the saturation magnetization and the coercive field with the temperature.

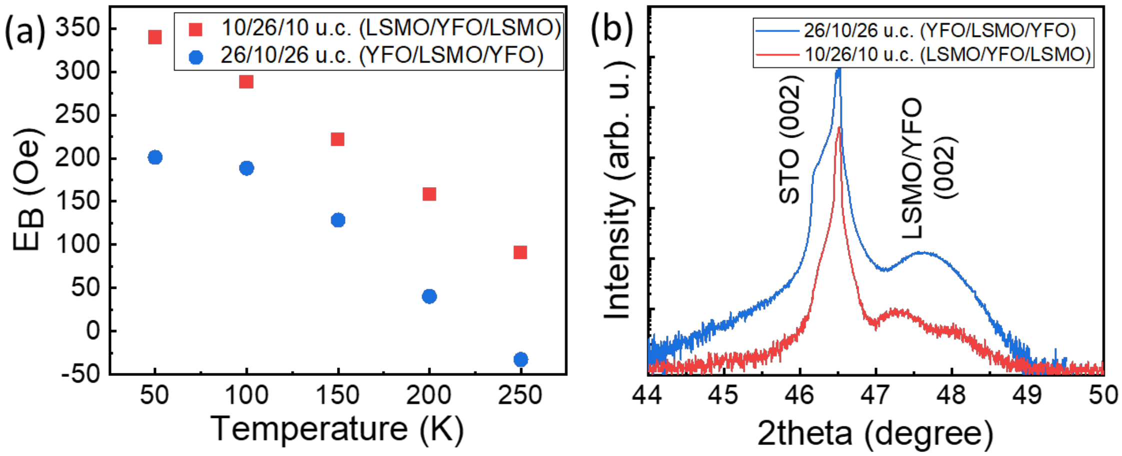

Figure S5 – Effect of layer order on EB for two stacks of three-layers grown on CrysTec STO (100) substrates. (a) Evolution of EB with the temperature, (b) Inset of the (002) peaks. The lower EB and T_C in the YFO/LSMO/YFO is attributed to higher roughness caused by the first YFO layer.

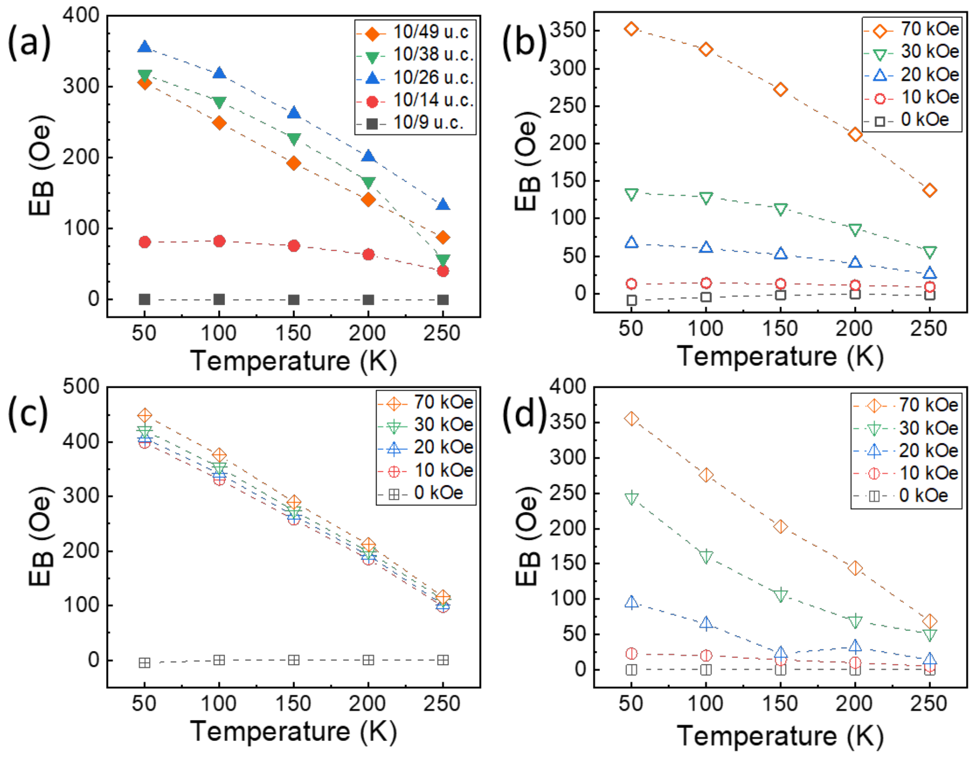

Figure S6 – (a) Training effect on bilayer samples shown in Figure 2(b) after a second field cooling at 10 kOe and 673 K. (b) Effect of different fields on EB while cooling the 10/26 u.c. bilayer sample at 370 K. (c-d) Evolution of EB under different field after annealing a 32 nm thick five-layer sample between its Curie and Néel temperature at 370K. (c) and (d) are measured using two different sequences, 0/70/10/30/20 kOe and 0/10/20/30/70 kOe respectively. Samples in (a), (b) and (c) are deposited on STO substrates from CrysTec Kristalltechnologie and the sample in (d) is deposited on STO substrate from MTI.

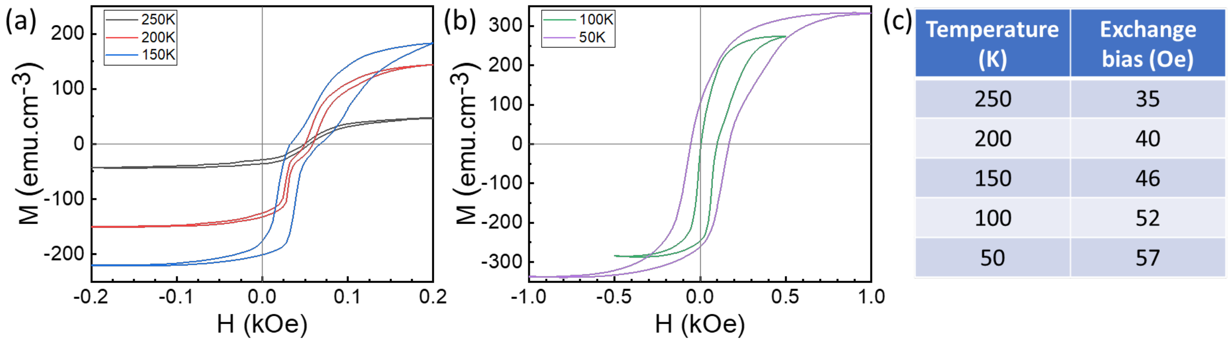

(c)

Temperature (K)	Exchange bias (Oe)
250	35
200	40
150	46
100	52
50	57

Figure S7 – (a-b) Minor magnetization loops at various temperature for the LSMO(10 u.c.)/STO(8 u.c.)/LSMO(10 u.c.)/YFO(26 u.c.) spin valve sample on CrysTec STO (100) cooled at 10 kOe from 673 K. (c) Table reporting EB vs temperature calculated from (a-b).

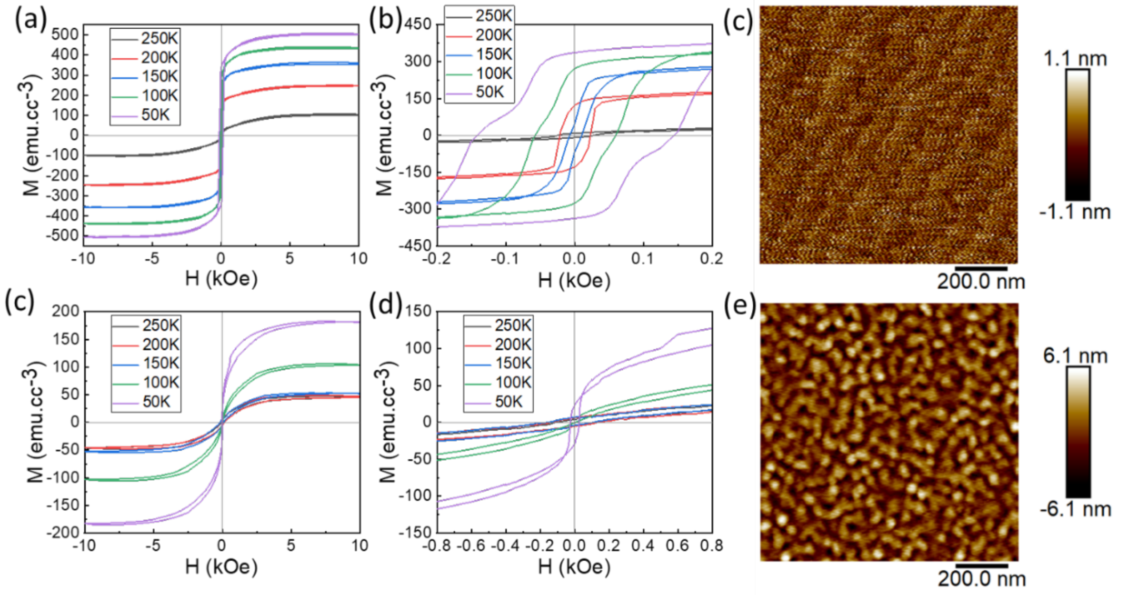

Figure S8 – Magnetic hysteresis loops performed on spin-valve stacks FC at 1T from 673 K deposited on (a-b) (100) STO from MTI and (c-d) (111) STO from MTI. (c) and (e) are AFM images of the stacks deposited on (100) and (111) STO respectively.

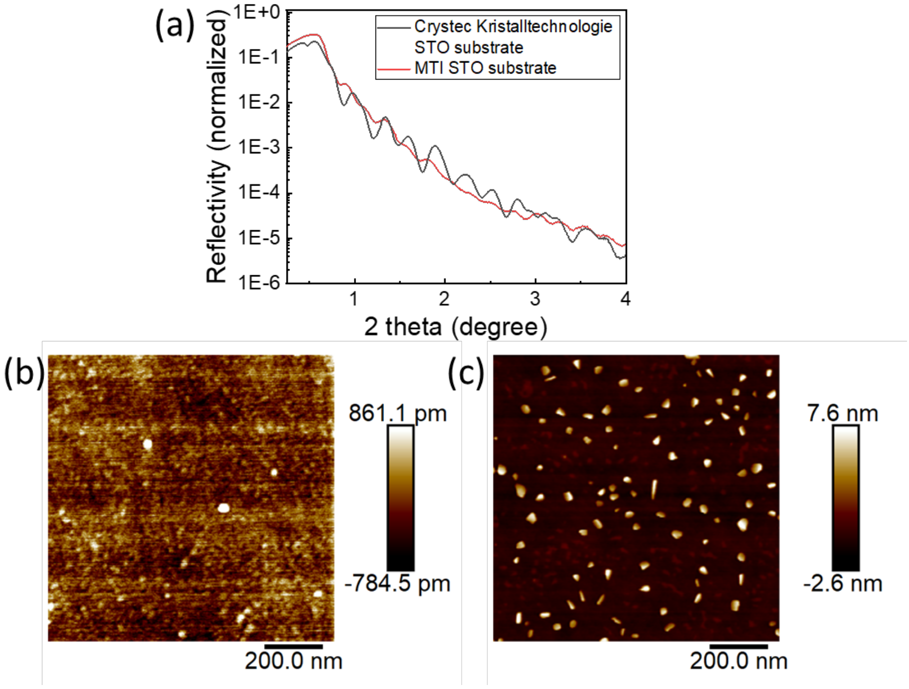

Figure S9 – (a) X-ray reflectivity measurements of the five-layers 10/26/10/26/10 u.c. samples deposited on CrysTec Kristalltechnologie and MTI STO substrates. (b) and (c) are AFM images of the bare STO substrates from CrysTec Kristalltechnologie and MTI, respectively.
